# Study on Miniaturized UHF Antennas for Partial Discharge Detection in High-Voltage Electrical Equipment

**DOI:** 10.3390/s151129434

**Published:** 2015-11-20

**Authors:** Jingcun Liu, Guogang Zhang, Jinlong Dong, Jianhua Wang

**Affiliations:** State Key Lab of Electrical Insulation and Power Equipment, Xi’an Jiaotong University, Xi’an 710049, China; E-Mails: liujingcun0523@163.com (J.L.); djl.1989@stu.xjtu.edu.cn (J.D.); jhwang@mail.xjtu.edu.cn (J.W.)

**Keywords:** equiangular spiral antenna, high-voltage electrical equipment, microstrip balun, partial discharge, ultra-high-frequency antenna

## Abstract

Detecting partial discharge (PD) is an effective way to evaluate the condition of high-voltage electrical equipment insulation. The UHF detection method has attracted attention due to its high sensitivity, strong interference resistance, and ability to locate PDs. In this paper, a miniaturized equiangular spiral antenna (ESA) for UHF detection that uses a printed circuit board is proposed. I-shaped, L-shaped, and C-shaped microstrip baluns were designed to match the impedance between the ESA and coaxial cable and were verified by a vector network analyzer. For comparison, three other types of UHF antenna were also designed: A microstrip patch antenna, a microstrip slot antenna, and a printed dipole antenna. Their antenna factors were calibrated in a uniform electric field of different frequencies modulated in a gigahertz transverse electromagnetic cell. We performed comparison experiments on PD signal detection using an artificial defect model based on the international IEC 60270 standard. We also conducted time-delay test experiments on the ESA sensor to locate a PD source. It was found that the proposed ESA sensor meets PD signal detection requirements. The sensor’s compact size makes it suitable for internal installation in high-voltage electrical equipment.

## 1. Introduction

The safety and reliability of high-voltage electrical equipment helps ensure the stable performance of a power system, but insulation breakdowns account for approximately 80% of total failures [1]. Therefore, it is important to assess, diagnose, and predict the insulation status of high-voltage electrical equipment. Partial discharge (PD) is low-energy ionization or a small electrical spark occurring in an insulating component that lacks permittivity and dielectric strength homogeneity. As those characteristics decline, the number and magnitude of the PD pulses increase rapidly, which may result in flashover and total breakdown. The presence of PD is a common symptom of insulation deterioration. Consequently, PD detection is a feasible way to avoid insulation degeneration. PD detection is now compulsory for judging the health and remaining life of high-voltage electrical equipment such as switchgear, transformers, and gas insulated switchgear (GIS) [2,3,4,5,6,7]. Several methods of PD detection and measurement have been developed and applied, such as high-frequency pulse current (HFPC) method; optical, chemical, and ultrasonic methods; radio frequency (RF) method; transient earth voltage (TEV) method and ultra-high frequency (UHF) method [8,9,10,11,12,13,14,15].

Among those methods, the UHF method is practical and effective due to its accuracy in locating PD sources and its convenience for online PD monitoring [16,17]. A key component of the UHF sensor is its antenna, which is important for acquiring and identifying UHF signals. UHF antennas can be either internal or external, depending on whether they are mounted inside or outside the high-voltage electrical equipment. External antennas are limited by their low sensitivity because of the shielding effect of the equipment casing and the interference or noise from the electromagnetism environment. This necessitates a close study of internal antennas. The width of a PD pulse could be as low as several nanoseconds or even sub-nanoseconds, so it is generally accepted that the UHF signal excited by PD lies in the range of 300 MHz to 3 GHz. This wide frequency range means that UHF internal antenna performance depends strongly on the antenna’s dimensions.

As a result, researchers have long been interested in optimizing the antenna’s broadband characteristics and minimizing antenna size. Li presented a novel compact multiband Hilbert fractal microstrip antenna for transformers, taking into account the frequency band and suitable size. The antenna was small, with a bandwidth of a few hundred MHz, but it also had very low gain [3]. Based on the designs of horn antennas, biconical log-periodic antennas, loop antennas, and dipole antennas, Kaneko built an improved dipole antenna and achieved high sensitivity [5]. Álvarez proposed optimized high-frequency current transformers (HFCT) and UHF sensors for online PD measurement [6]. Robles chose four types of antennas, including two monopoles, a zigzag antenna, and a log-periodic antenna, to measure PDs and contrasted their differing frequency behaviors. That research showed that a monopole of 5 cm was the best option [8]. Ye manufactured a multiband antenna using loop-antenna theory and a meandering technique. It worked in bandwidth ranges of 480–520, 800–850, and 1100–1200 MHz [12]. Li designed an internal two-arm equiangular spiral antenna (ESA) for gas insulated switchgear that greatly improved UHF antenna performance, but its impedance transformer made it unsuitably large [10].

To summarize, the existing literature covers UHF antennas in GIS, transformers, switchgears and XLPE cables. However, antennas with known wideband performance, such as log-periodic or spiral antennas, are always significantly large in size. For high-voltage electrical equipment, an internal UHF antenna is required for both its performance and its size. In the research for this paper, a sensor comprising a dual-arm planar ESA and a miniaturized microstrip balun was fabricated and tested. Its design was based on printed circuit board (PCB) technology. In addition, three other UHF antennas—a microstrip patch antenna (MPA), a microstrip slot antenna (MSA), and a printed dipole antenna (PDA)—were also fabricated and tested to compare them with the ESA sensor. Critical performance parameters in the UHF range were optimized through simulation and verified by a vector network analyzer (VNA). Using a standard EM field modulated in a gigahertz transverse electromagnetic (GTEM) cell, the antenna factors (AFs) of those sensors, which describe receiving characteristics to determine electric field strength, were measured and fitted. Based on the PD quantity reference provided by the traditional IEC 60270 method, and an artificial defect model as a PD source, we tested the performances of the proposed sensors and their time-delay characteristics in locating a PD source.

This paper has five parts: Section 1 is the introduction; Section 2 explains the design and optimization of ESA, its impedance balun, and the other three antennas; Section 3 describes the measurement of AFs in a GTEM cell; Section 4 shows the UHF signal detection of the antennas; and Section 5 presents the conclusions.

## 2. Design of the UHF Antennas

Various components must occupy the compact inner space of high-voltage electrical equipment. Because of this, internal UHF sensors should meet the following requirements: compact dimensions, strong anti-interference ability, good directivity, and high gain. A UHF sensor should work in the frequency range of 300 MHz to 3 GHz. Frequency independent antennas, such as the Archimedean spiral antenna, the ESA, and the log-periodic dipole antenna, which perform the same way at every frequency, are a perfect choice for this application. However, the disadvantage of most frequency independent antennas is that they are too large to be installed inside high-voltage electrical equipment, so much effort has been made to reduce their size. The ESA, developed in the 1950s, is an ideal type of frequency independent antenna. When requirements are not excessively restrictive, the ESA can accommodate a wide operational bandwidth while being reduced to a reasonable size. Therefore, an ESA was chosen as the design prototype in this work. MPA, MSA, and PDA were also built and tested for comparison.

In practice, the ESA often suffers from lack of an appropriate planar feeding way. A balun (balance–unbalance) element is used to change a balanced feed to an unbalanced feed; *i.e.*, the ESA to a coaxial cable. Another function of the balun is impedance conversion. A typical balun is designed vertically, but that makes it too long to install in the limited internal space of high-voltage electrical equipment. In this paper, three kinds of modified horizontal baluns were proposed to significantly reduce the overall size of the ESA. The performance of the designed ESA with the horizontal baluns were measured and studied by a VNA.

### 2.1. Design of a Dual-Arm ESA

To create an ESA, in the polar coordinates system the equation of one spiral curve should be written as:

(1) $$\rho =\rho_{0}e^{a\varphi }\;\rho \leq \rho_{max}$$

where *ρ* is the polar radius, which is the radial distance between the center of the spiral and the point at the polar angle *φ*, such that *ρ*_0_ is the radial distance at *φ* = 0, and *ρ*_max_ refers to the radial distance at *φ* = *φ*_max_. The rate of wrapping is determined by the constant *a*. The equiangular spiral has the property that at any two points the angles between the tangent and radial vectors are equal. In fact, the wrap angle *α* can be related to *a* by:

(2) $$\tan\alpha =\frac{1}{a}$$

For a certain frequency, when the arm length from vertex to edge is equal to wavelength *λ*, the corresponding radial length is approximately $\frac{\lambda }{4}$. The relationship between wavelength and frequency is:

(3) $$\lambda =\frac{c}{f}$$

where *c* is the speed of light. Therefore, the inner and outer radii of the ESA can be calculated for the bandwidth of interest. For example, *ρ*_0_ is defined by the superior frequency limit:

(4) $$\rho_{0}=\frac{\lambda_{min}}{4}$$

The radial distance *ρ*_0_ is 2.5 mm because the working band is up to 3 GHz. The outer radius *ρ*_max_ is defined by the inferior frequency limit:

(5) $$\rho_{max}=\frac{\lambda_{max}}{4}$$

**Figure 1:**
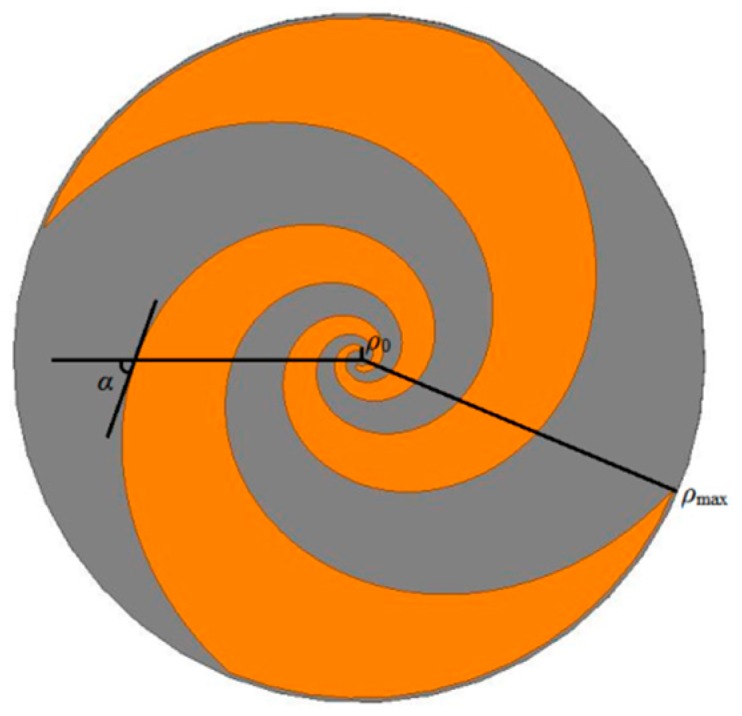
Geometry of a dual-arm ESA.

Because the minimum working frequency is 500 MHz, *ρ*_max_ should be 150 mm, but this dimension was reduced to 62 mm to meet the installation requirements of the sensor. In other words, low-frequency performance was sacrificed to its practical application. With the above curve drawn, rotating it repeatedly by 90° formed the edges of two arms of the ESA, which made the ESA a self-complementary structure (Figure 1).

Other determined parameters of ESA are listed in Table 1.

**Table 1:** Parameters of the designed ESA.

Wrap Angle	Rotation Angle	Substrate Thickness	Relative Permittivity
70°	3 π	1.6 mm	4.4

**Figure 2:**
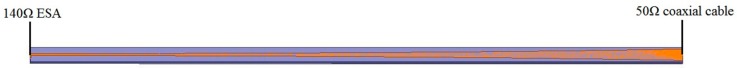
Structure of the exponential asymptote microstrip balun.

In practice, the impedance was approximately 140 Ω, according to simulation and measurement [16,17]. The antenna was connected to a 50 Ω coaxial cable, so an exponential asymptote microstrip balun was designed to provide broadband matching. Figure 2 shows the structure of the balun. Its total length was 220 mm. The width at the feed point was 0.5 mm; the width at the other end was 4.5 mm.

**Figure 3:**
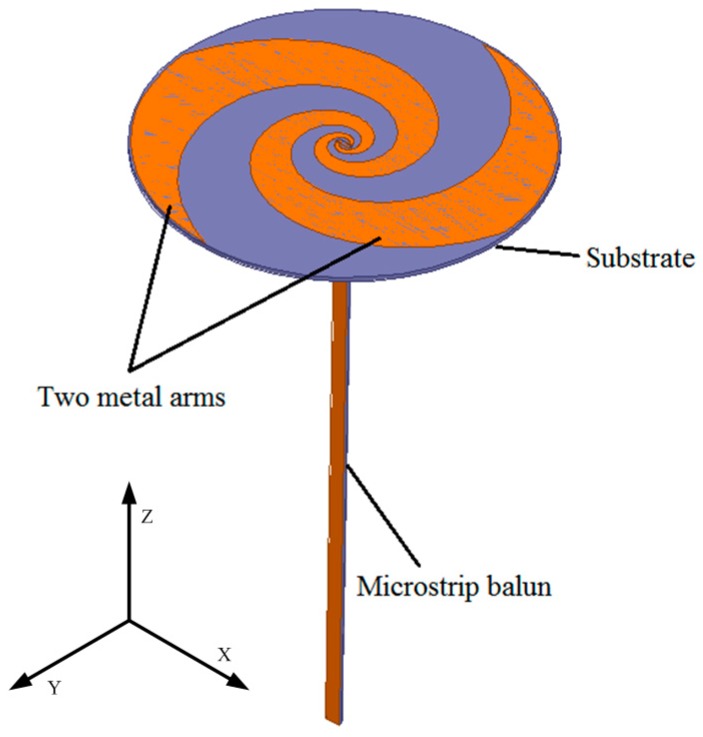
Overall design of the dual-arm ESA and the vertical microstrip balun.

Initially, the microstrip balun was connected to ESA vertically, as shown in Figure 3.

**Figure 4:**
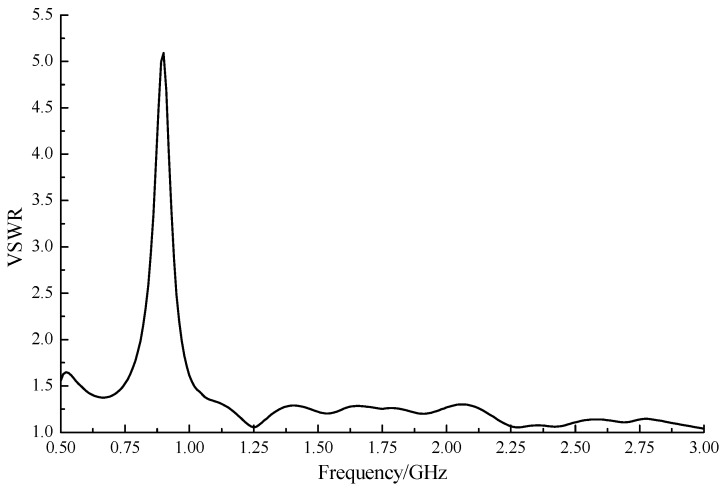
Voltage standing-wave ratio (VSWR) simulation results of the experimental ESA.

We conducted a thorough simulation using the three-dimensional EM simulation software ANSYS HFSS. A linear frequency sweep of 500 MHz to 3 GHz and a 10-MHz step size was configured in the simulation. The results of the key parameters of the designed ESA are shown in Figure 4 through Figure 6.

The VSWR indicates the impedance match between an antenna and a feed line. The smaller the VSWR is, the better the match. In normal applications, an antenna works well in a frequency band where the VSWR is less than 2. In Figure 4, the frequency band whose VSWR is less than 2 lay almost in the whole sweep range, except for the 820 MHz to 960 MHz portion.

The VSWRs of the other frequencies were close to 1.25 and remained stable, which indicated little reflection loss and decent antenna performance. The working bandwidth was 2.36 GHz and the relative bandwidth was up to 94.4%. It meant that even though low-frequency performances were sacrificed to reduce the size of the designed ESA, that antenna could still meet the signal detecting requirement.

Input impedance is the ratio of the voltage to the current at an antenna input [18]. Because 50 Ω coaxial cable is widely used, it is required that the real input impedance approach 50 Ω and the imaginary impedance be as near to 0 as possible.

**Figure 5:**
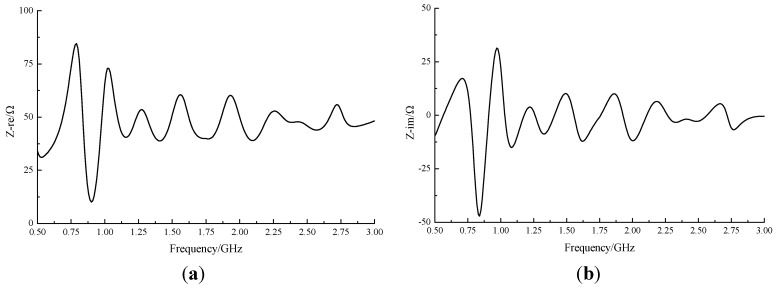
Impedance simulation results of the ESA: (**a**) real impedance; (**b**) imaginary impedance.

**Figure 6:**
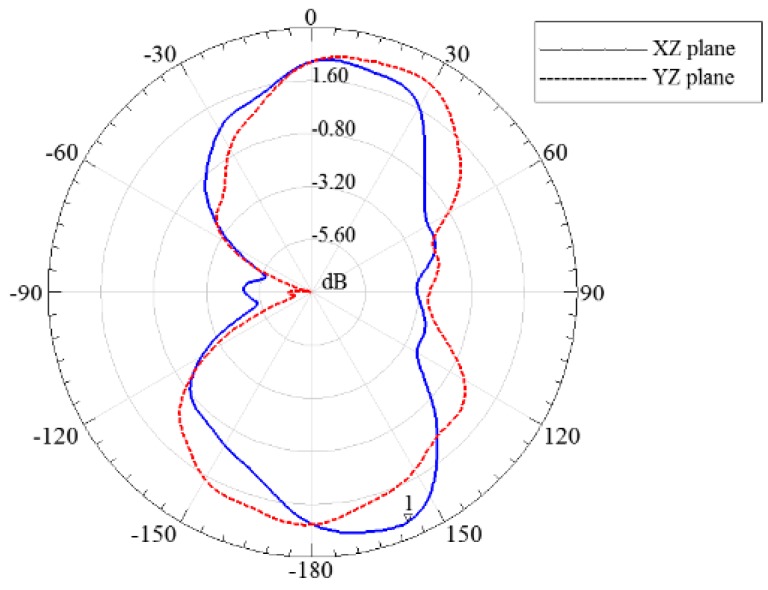
Radiation pattern simulation results of the ESA.

Figure 5 shows impedance matching conditions in our tests. The real input impedance fluctuated around 50 Ω and the imaginary input impedance fluctuated around 0. The scope of the fluctuation under 1 GHz was approximately 35 Ω; above 1 GHz the scope was within 10 Ω. Thus, the exponential asymptote microstrip balun did provide wideband impedance matching for the ESA. Gain describes the ratio of the power density of an actual antenna to an ideal radiating element in the same position under the same conditions. Realized gain is the term used when reflection loss due to impedance is taken into account. It is the ratio of power radiated to the power input of the antenna. Generally speaking, the higher the realized gain, the stronger the signal a UHF antenna receives. The directivity of an antenna refers to the distribution of the energy it radiates [19]. A radiation pattern is used to judge the directivity of an antenna in practical applications. Figure 6 shows the radiation pattern of the XZ and YZ planes at a center frequency of 1.75 GHz. At marker 1, the peak realized gain was up to 3.24 dB. The results also show the irregularity of the ESA’s directivity, which means it is a unidirectional antenna. As a result, the angle and position of the ESA’s installation must be considered in experimental and practical applications.

### 2.2. Design and Optimization of the Balun

**Figure 7:**
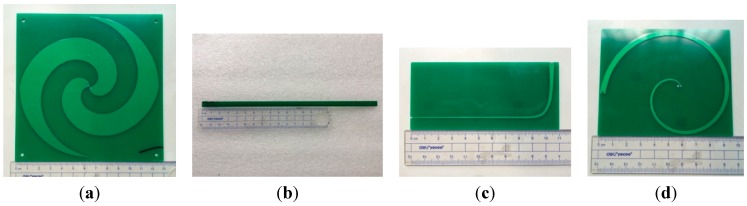
Manufactured prototype dimensions of the equiangular spiral antenna (ESA) and microstrip baluns. (**a**) ESA; (**b**) I-shaped balun; (**c**) L-shaped balun; (**d**) C-shaped balun.

Clearly, an ESA with a long vertical balun performs well but is oversized. To reduce the size of the designed sensor for an internal installation, three main approaches are presented in the following. The prototype ESA is shown in Figure 7a, and three types of microstrip baluns are shown in Figure 7b through Figure 7d.

(1) Horizontal Balun

As discussed above, a vertical balun adds unwanted depth and hinders reliable installation. The first improvement approach was to simply place the same exponential asymptote balun, called an I-shaped microstrip balun, horizontally, as shown in Figure 7b. This slightly increased the length of the whole sensor, but clearly reduced the 3D space the sensor occupied.

(2) Deformed Balun

With the metal ground plate patch on one side of the substrate remaining unchanged, the second approach deformed the shape of the microstrip balun on the other side of the substrate. As shown in Figure 7c, the L-shaped microstrip balun was designed by bending the asymptote line 90°. Figure 7d shows an innovative spiral balun called a C-shaped microstrip balun. The two edges of the line were based on an Archimedean spiral, with equal widths at the feed point and the other end. The L-shaped and C-shaped baluns were shorter than the original balun.

(3) Integrated Balun

Ideas to integrate feed into antennas are provided in previous studies. Constructions similar to the I-shaped and C-shaped baluns have been designed and tested [20,21,22,23]. Integrating baluns into a spiral antenna renders the entire system two-dimensional. Results show that antennas fed by this method work well in the ultra-wideband (UWB) range of 3.1 GHz to 10.6 GHz. However, for the UHF band an integrated balun could occupy more space and be difficult to manufacture. Therefore, in this study that method is not considered.

**Figure 8:**
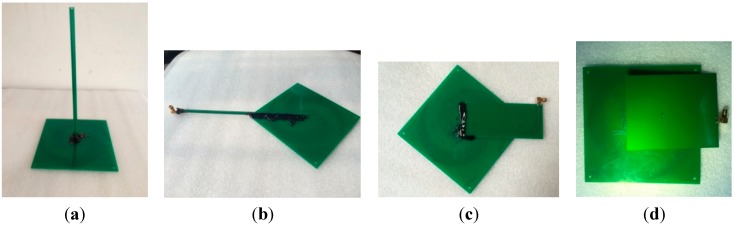
Connections of the ESA and baluns. (**a**) vertically; (**b**) with an I-shaped balun; (**c**) with an L-shaped balun; (**d**) with a C-shaped balun.

Figure 8 indicates how the ESA and baluns connect to the back of the antenna. Obviously, a sensor made of the ESA and a vertical balun occupies too much space. The modified baluns can be placed on almost the same plane as the ESA, which is desirable for installation and fixation.

**Figure 9:**
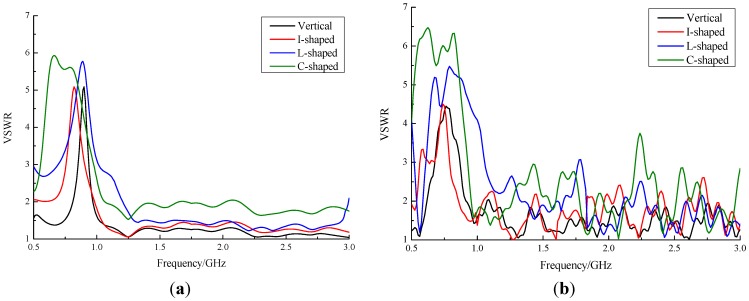
Comparison results of four types of baluns: (**a**) simulation; (**b**) VNA measurement.

To find the best of the optimized baluns, simulations and measurements were conducted. The simulation configuration was also a linear sweep of 500 MHz to 3 GHz with a step size of 10 MHz. VSWRs of four antennas were measured by an Agilent E5061B VNA. Figure 9 shows the comparison results of the four types of baluns. Under 1 GHz, simulation results were very similar to those of the real performance. Over 1 GHz, measured VSWRs were higher due to the limitations of the VNA and the high-frequency loss of coaxial cable. In general, broadband performance of the three optimized baluns was similar to that of the original vertical balun; the I-shaped balun was the closest. For the original vertical balun, the working band where the VSWR was less than 2 was 500–600 MHz and 800 MHz–3 GHz, and bandwidth was 2.25 GHz, which suggests that deviation from simulation was less than 6%. Meanwhile, the I-shaped balun performed slightly worse but much like the vertical type. Its working band included 500–550 MHz, 850–1050 MHz, and 1.15–3 GHz; bandwidth was 2.1 GHz and relative bandwidth was 84%. The VSWR of the L-shaped and C-shaped baluns at low frequencies increased substantially. Therefore, the ESA with the I-shaped microstrip balun was chosen for the following experiments.

### 2.3. Design of Other Antennas

In the field of communications, many types of antennas that work in the UHF band, such as the microstrip antenna, have been used. Previous studies on UHF detection of PD have long been focused on these common types of antennas. Their main advantages are their small size, design simplicity, robustness, and convenient adaptation to mass production using PCB technology. Therefore, in this paper three miniaturized antennas are designed and printed on dielectric substrates to compare with the ESA and determine the optimal antenna for PD measurement.

**Figure 10:**
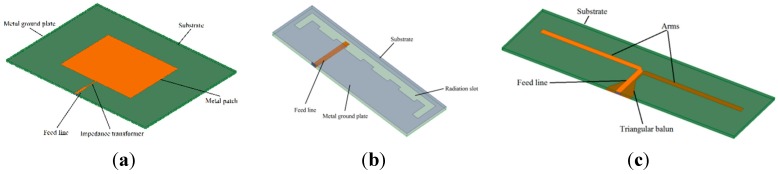
Structure of 3 common antennas: (**a**) MPA; (**b**) MSA; (**c**) PDA.

Figure 10a illustrates the structure of an MPA that has been widely used in recent years. It is fabricated by etching the antenna element pattern on a metal trace. The trace is bonded to an insulating dielectric substrate, with a continuous metal layer bonded to the opposite side of the substrate to form a ground plane. For impedance matching with a coaxial cable, a one-quarter wavelength impedance transformer is added. Similarly to an MPA, as shown in Figure 10b, an MSA is made by slotting on its metal ground plate. In the illustrated design, a feed and meandering method have been used to aid miniaturization. A PDA is a deformation of a half-wave dipole antenna. The version shown in Figure 10c has a metal arm on each side of the dielectric substrate. Because it is a balanced structure, this PDA must incorporate a triangular balun to balance the antenna with the coaxial cable.

Several critical parameters of the ESA and our three final designed antennas are listed in Table 2, with the geometry and performance optimized by simulation. Results indicate that the ESA had a significant advantage in bandwidth and gain, while the MSA and PDA, with their small geometry and good directivity, are easy to install. Overall, each of the four antennas meets the requirements of PD detection.

**Table 2:** Parameters of four types of UHF antennas.

Type of Antenna	Geometry in mm	Working Band in MHz	Directivity	Peak Realized Gain/dB
ESA	124 × 124 × 1.6	500–550	Poor	3.24
		850–1050		
		1150–3000		
MPA	188 × 138 × 1.6	920–1020	Poor	−1.54
MSA	100 × 30 × 1.6	961–979	H plane good	−0.17
PDA	140 × 40 × 1.6	908–1029	H plane omnidirectional	1.40

## 3. Calibration of the Antenna Factor

To obtain a quantitative relationship between measured electric field strength and antenna output voltage, antenna calibration is required. The antenna factor is defined mathematically as:

(6) $$AF=\frac{E_{incident}}{V_{received}}$$

It measures the received voltage in the presence of an electric field. For PD detection applications, once the AF of an antenna is determined, electric field strength on the reference plane excited by a PD source can be calculated. That helps to judge the insulation condition of the target equipment. In this study, antenna calibration took place in the standard electric field generated inside a GTEM cell. That device is a large, tapered coaxial structure, with transverse electromagnetic waves excited around the core board. A GTEM cell provides an EM field with great uniformity and reproducibility in a desired region of space. Using the uniform electric field within a workspace under different frequencies, we conducted experiments in a GTEM cell to measure AFs.

### 3.1. Experimental Setup

**Figure 11:**
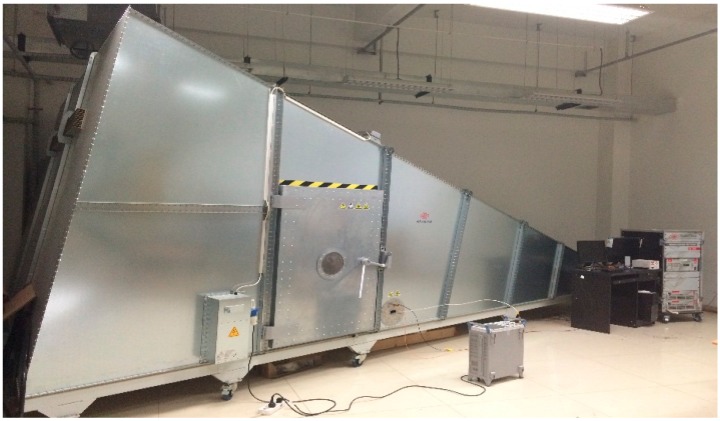
Experimental setup for calibration of AFs.

The experiment configuration is shown in Figure 11. The signal generator, connected to the GTEM cell as excitation source, modulated the needed frequencies and field strengths of the EM field. The four types of antennas were placed in the center zone of the test cell (one-third of the septum height). In that area of the GTEM cell, the electric field vector is vertical and the EM wave should be considered as linearly polarized. Therefore, to achieve the maximum received signal and the least loss from polarization mismatch, the electric field vectors of the antennas should match. For horizontally linearly polarized antennas like MPAs, MSAs, and PDAs, the E plane is positioned in parallel with the vertical electric field vector. For circularly polarized antennas like ESAs, the orientation could be flexible as long as the antenna axis is horizontal, so during the experiment, all four antennas were oriented vertically. Their received signals were output to an R&S ESR EMI test receiver through a 50 Ω coaxial cable. Before the signal generator was turned on, a modulation range of frequencies was set. During the experiment, the output signals of UHF antennas were recorded by the EMI receiver at every frequency.

### 3.2. Experimental Result and Antenna Factor Determination

**Figure 12:**
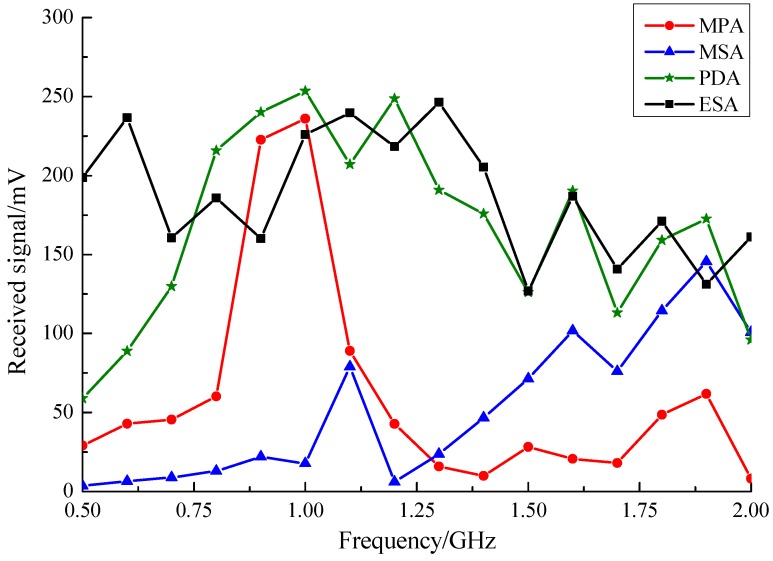
Frequency sweep results from testing in the GTEM cell.

Figure 12 shows the frequency sweep (500 MHz to 2 GHz, step size 100 MHz) results of the ESA and three other sensor prototypes. Results suggest that the amplitude of the signal the ESA detected was over 150 mV and remained stable in the sweep range. For the PDA, the amplitude of the output signal at frequencies below 700 MHz was low, while beyond 700 MHz the amplitude increased to almost the same as that of the ESA. For the MPA, the signal amplitude was highest near the 970 MHz center frequency, but lower than 50 mV at other frequencies. For the MSA, the signal amplitude beyond 1 GHz grew higher, while it was very low at lower frequencies.

Antenna factor (AF) expresses the relationship between an output signal and electric field strength, which is a function of frequency (*f*, GHz). We based our calibration of AFs on previous experimental data. Five times polynomial fitting was done by delineating a scatterplot of calculated AF values. Table 3 shows the coefficients of fitting Equation (7):

(7) $$AF=B_{5}f^{5}+B_{4}f^{4}+B_{3}f^{3}+B_{2}f^{2}+B_{1}f+C$$

**Table 3:** Coefficients of the fitting equation of AFs.

Type of Sensor	*C*	*B_1_*	*B_2_*	*B_3_*	*B_4_*	*B_5_*
MPA	−2781.3	21,840	−53,003	55,908	−26,426	4602.6
MSA	30,694	−119,108	182,850	−135,273	48,086	−6592.8
PDA	878.7	−2298.6	2033.5	−475.5	166.9	67.0
ESA	−663.7	3427.1	−6121.1	5132.7	−2040.7	311.2

**Figure 13:**
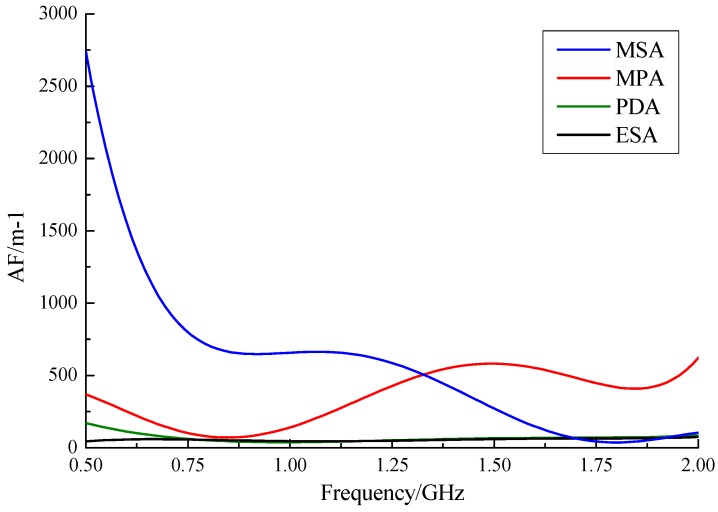
Calibration curves of antenna factors for four antenna types.

As shown in Figure 13, the AF of the proposed ESA sensor was the lowest and stabilizes at approximately 50 in the whole band, while the AFs of others were higher and fluctuated. The ESA was the most sensitive antenna.

## 4. UHF Detection Experiments

**Figure 14:**
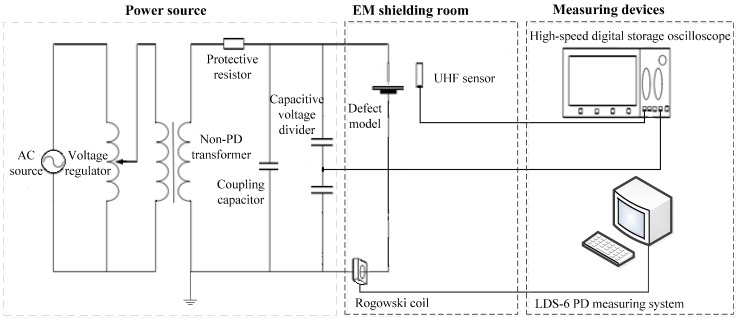
The PD experiment platform.

**Figure 15:**
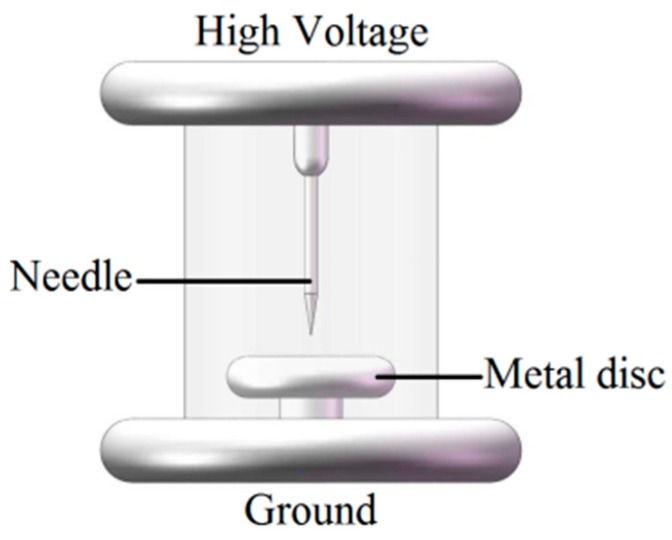
The artificial defect model.

To study the performances of the antennas developed in this paper, a PD experiment platform was set up in an EM shielding room, as illustrated in Figure 14. The high-voltage power source of the circuit was provided by a non-PD testing transformer. To simulate a PD source, we designed a needle-plane electrode system (Figure 15) as a typical artificial defect model. An LDS-6 PD measuring system was adopted to measure the PD magnitude, which was based on the IEC 60270 standard and had a resolution of 1 pC. A Tektronix DPO7104 high-speed digital storage oscilloscope was used as the signal recorder, with an input channel bandwidth of 1 GHz, a sampling rate of 20 GHz, and a memory depth of 32 M.

### 4.1. Comparison Experiment of PD Detection for the Four Sensors

**Figure 16:**
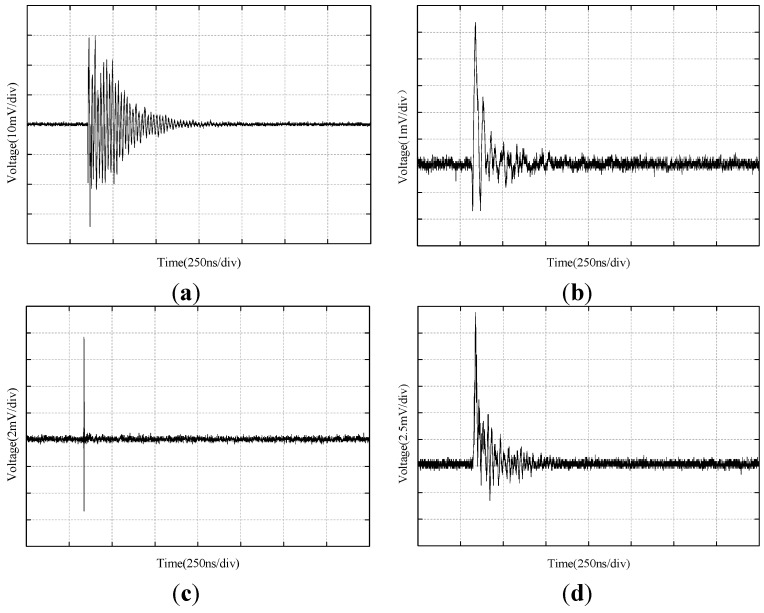
UHF waveforms of the PD comparison experiment: (**a**) ESA; (**b**) MPA; (**c**) MSA; (**d**) PDA.

In the experiment, the four antennas were placed 2 m from the defect model and connected to an oscilloscope through coaxial cable to display the output signal. When the discharge quantity on the LDS-6 system stabilized, the UHF signal sample of each antenna on the oscilloscope was recorded. The reference discharge quantity was 320 pC; the collected PD UHF single-cycle waveforms of the four sensors are shown in Figure 16. The signal magnitude of the ESA was up to approximately 35 mV and the waveform detail was clear. Moreover, the measured sensitivity of the ESA reached 16 pC when the background noise was 8 pC. The signal magnitude of the MPA was approximately 4.5 mV, so it was hard to detect low-energy PD or PD when background noise was high. The signal magnitude of the MSA was approximately 8 mV, but its waveform was difficult to restore effectively due to its narrow bandwidth. For the PDA, the signal magnitude was approximately 15 mV, but the symmetry of its signal was poor, because the negative part of the waveform was not as clear as the positive part.

### 4.2. Time-Delay Experiment

A triangulation method is widely used to detect PD locations [24,25,26]. By measuring the delay time of a UHF pulse received by two sensors in different locations, the distance between the two sensors can be calculated by:

(8) L=ct

where *c* is the speed of light and *t* is the measured delay time. In practical applications it usually takes four sensors to determine a PD source location in 3D space using the following equations:

(9) $$\{\begin{array}{c} \begin{array}{c} {\left(x-x_{1}\right)}^{2}+{\left(y-y_{1}\right)}^{2}+{\left(z-z_{1}\right)}^{2}={\left[cT\right]}^{2}\\ {\left(x-x_{2}\right)}^{2}+{\left(y-y_{2}\right)}^{2}+{\left(z-z_{2}\right)}^{2}={\left[c(T+t_{12})\right]}^{2} \end{array}\\ {\left(x-x_{3}\right)}^{2}+{\left(y-y_{3}\right)}^{2}+{\left(z-z_{3}\right)}^{2}={\left[c(T+t_{13})\right]}^{2}\\ {\left(x-x_{4}\right)}^{2}+{\left(y-y_{4}\right)}^{2}+{\left(z-z_{4}\right)}^{2}={\left[c(T+t_{14})\right]}^{2} \end{array}$$

where (*x*, *y*, *z*) are the coordinates of the source, (*x_i_*, *y_i_*, *z_i_*) are the coordinates of sensor *i*, *T* is the time from source to the first sensor, and *t_1i_* is the delay time of sensor *i* and the first sensor.

**Figure 17:**
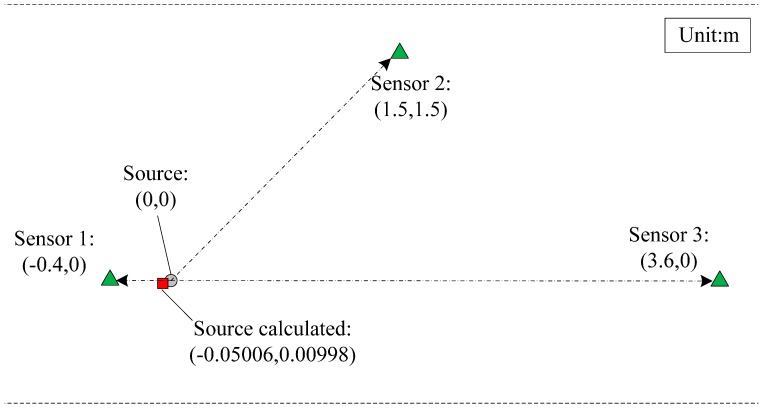
Schematic diagram of the positions of the source and the three sensors.

**Figure 18:**
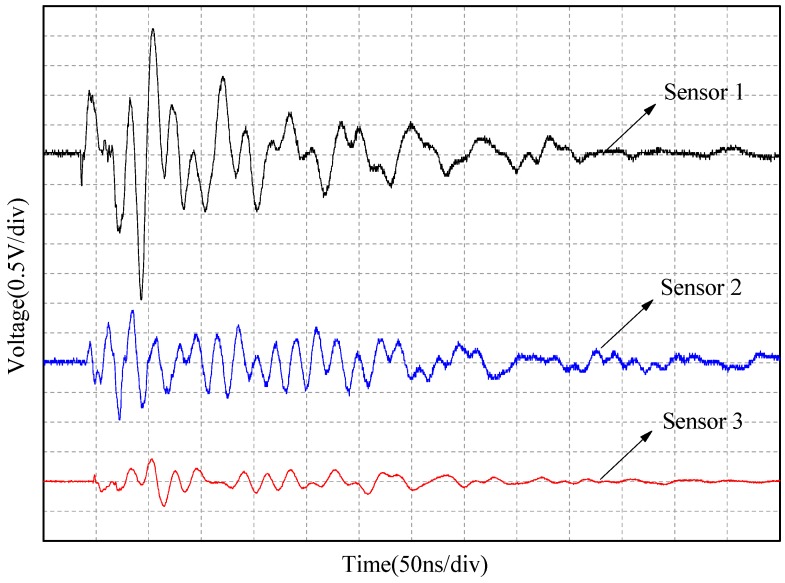
Time-delay experiment waveforms of three ESA sensors.

To determine a PD source location in 2D space, we placed 3 ESA sensors at different locations and connected them to three channels of the oscilloscope by coaxial cables of identical lengths. Figure 17 shows the positions of the three ESA sensors and the artificial defect model. To prove the feasibility and accuracy of this method using the proposed antenna, the PD source position was calculated by Equation (9) and compared with the preset position. The collected PD UHF waveforms of the three sensors are shown in Figure 18. It can be seen that the delay time between Antennas 1 and 2 was 6 ns and between Antennas 1 and 3 was 11 ns. Thus, the calculated PD source coordinate was approximately (−0.05, 0.01), which means the deviation from the actual source location was only 5 cm or less.

## 5. Conclusions

This study focused on the design and optimization of internal antennas for UHF detection of PDs in high-voltage electrical equipment. The proposed dual-arm ESA satisfied the requirements of both broadband performance and compact size. An MPA, an MSA, and a PDA were also developed and tested for comparison. A GTEM cell was used for calibrating AFs. A PD detecting experiment platform was set up to test the performances of the proposed antennas. A time-delay experiment to locate the PD source was also conducted. The results of the study can be summarized as follows:

(1) The proposed antenna was designed using the basic configuration of an ESA, while its outer radius was reduced to 62 mm to meet the strict installation requirements of an internal sensor. The key parameters were determined through simulation. It was found that an ESA can work in the UHF band range with high realized gain and good impedance matching, although its directivity is not ideal and should be considered.
(2) An I-shaped balun, an L-shaped balun, and a C-shaped balun are attempted to further reduce the size of the entire sensor. A VSWR measurement on a VNA was conducted. The I-shaped horizontal balun performed like the original vertical type with a wide working band (500–550 MHz, 850–1050 MHz, and 1.15–3 GHz); the 2.1 GHz bandwidth was the best option.
(3) An MPA, an MSA, and a PDA were designed, and their performances were also analyzed and optimized. The main differences between the ESA and the other three types of sensors lay in bandwidth and the AF. The result of calibration in the GTEM cell indicated that the AF of the proposed ESA sensor was the lowest and most stable, while the AFs of the others were higher and fluctuated.
(4) The experiment on UHF detection of PD by the four types of sensors showed that the performance of the ESA sensor surpassed those of the others. The magnitude of signal received by the ESA sensor was the largest, and the shape and details of the PD waveform were clearly visible on the oscilloscope. An experiment using a triangulation method to locate a PD source using the proposed ESA was also conducted. It was found that the proposed ESA revealed a PD source location with a precision of 5 cm.

## References

[1] Sarkar B., Mishra D.K., Koley C., Roy N.K. Microstrip patch antenna based UHF sensor for detection of partial discharge in high voltage electrical equipments. Proceedings of the Annual IEEE India Conference.

[2] Yao C., Chen P., Huang C., Chen Y., Qiao P. (2013). Study on the application of an ultra-high-frequency fractal antenna to partial discharge detection in Switchgears. Sensors.

[3] Li J., Jiang T., Cheng C., Wang C. (2013). Hilbert fractal antenna for UHF detection of partial discharges in transformers. IEEE Trans. Dielectr. Electr. Insul..

[4] Sarathi R., Koperundevi G. (2009). Investigation of partial discharge activity of single conducting particle in transformer oil under DC voltages using UHF technique. IET Sci. Meas. Technol..

[5] Kaneko S., Okabe S., Yosh M., Muto H., Nishida C., Kamei M. (2009). Detecting characteristics of various type antennas on partial discharge electromagnetic wave radiating through insulating spacer in gas insulated switchgear. IEEE Trans. Dielectr. Electr. Insul..

[6] Álvarez F., Garnacho F., Ortego J., Sánchez-Urán M.Á. (2015). Application of HFCT and UHF sensors in on-line partial discharge measurements for insulation diagnosis of high voltage equipment. Sensors.

[7] Wang L., Fang N., Wu C., Qin H., Huang Z. (2014). A fiber optic PD sensor using a balanced Sagnac interferometer and an EDFA-Based DOP Tunable fiber ring laser. Sensors.

[8] Robles G., Sanchez-Fernandez M., Albarracin S.R., Rojas-Moreno M.V., Rajo-Iglesias E., Martinez-Tarifa J.M. (2013). Antennas parameterization for the detection of partial discharges. IEEE Trans. Instrum. Meas..

[9] Hoshino T., Maruyama S., Ohtsuka S., Hikita M., Wada J., Okabe S. (2012). Sensitivity comparison of disc- and loop-type sensors using the UHF method to detect partial discharges in GIS. IEEE Trans. Dielectr. Electr. Insul..

[10] Li T., Rong M., Zheng C., Wang X. (2012). Development simulation and experiment study on UHF partial discharge sensor in GIS. IEEE Trans. Dielectr. Electr. Insul..

[11] Piccin R., Mor A., Morshuis P., Girodet A., Smit J. (2015). Partial discharge analysis of gas insulated systems at high voltage AC and DC. IEEE Trans. Dielectr. Electr. Insul..

[12] Hai-feng Y., Xiu-chen J., Yong Q., Ge-hao S., Yue D. (2014). Development of multi-band ultra-high-frequency sensor for partial discharge monitoring based on the meandering technique. IET Sci. Meas. Technol..

[13] Muslim J., Susilo A., Nishigouchi K., Kozako M., Hikita M., Arief Y.Z., Khayam U., Suwarno Enhanced bowtie UHF antenna for detecting partial discharge in gas insulated substation. Proceedings of the 48th International Universities’ Power Engineering Conference.

[14] Lopez-Roldan J., Tang T., Gaskin M. (2008). Optimisation of a sensor for onsite detection of partial discharges in power transformers by the UHF method. IEEE Trans. Dielectr. Electr. Insul..

[15] Tenbohlen S., Denissov D., Hoek S., Markalous S.M. (2008). Partial discharge measurement in the ultra high frequency (UHF) range. IEEE Trans. Dielectr. Electr. Insul..

[16] Gao W., Ding D., Liu W., Huang X. (2013). Analysis of the intrinsic characteristics of the partial discharge induced by typical defects in GIS. IEEE Trans. Dielectr. Electr. Insul..

[17] Pinpart T., Judd M.D. Experimental comparison of UHF sensor types for PD location applications. Proceedings of the IEEE Electrical Insulation Conference.

[18] McFadden M., Scott W.R. (2007). Analysis of the equiangular spiral antenna on a dielectric substrate. IEEE Trans. Antennas Propag..

[19] Fu W., Lopez E.R., Rowe W.S.T., Ghorbani K. (2010). A planar dual-arm equiangular spiral antenna. IEEE Trans. Antennas Propag..

[20] Eubanks T.W., Chang K. (2010). A compact parallel-plane perpendicular-current feed for a modified equiangular spiral antenna. IEEE Trans. Antennas Propag..

[21] Mao S.-G., Yeh J.-C., Chen S.-L. (2009). Ultrawideband circularly polarized spiral antenna using integrated balun with application to time-domain target detection. IEEE Trans. Antennas Propag..

[22] Chen T.-K., Huff G.H. (2011). Stripline-fed Archimedean spiral antenna. IEEE Antennas Wirel. Propag. Lett..

[23] Veysi M., Kamyab M. (2011). Bandwidth enhancement of low-profile PEC-backed equiangular spiral antennas incorporating metallic posts. IEEE Trans. Antennas Propag..

[24] Hou H., Sheng G., Jiang X. (2013). Robust time delay estimation method for locating UHF signals of partial discharge in substation. IEEE Trans. Power Deliv..

[25] Markalous S., Tenbohlen S., Feser K. (2008). Detection and location of partial discharges in power transformers using acoustic and electromagnetic signals. IEEE Trans. Dielectr. Electr. Insul..

[26] Sinaga H.H., Phung B.T., Blackburn T.R. (2012). Partial discharge localization in transformers using UHF detection method. IEEE Trans. Dielectr. Electr. Insul..

